# Heterogenous Mental Health Impacts of a Forced Relocation: The Red Zone in Christchurch (New Zealand)

**DOI:** 10.1002/hec.70004

**Published:** 2025-06-21

**Authors:** Thoa Hoang, Ilan Noy, Thinh Le Van

**Affiliations:** ^1^ Thuy Loi University Hanoi Vietnam; ^2^ Victoria University of Wellington Wellington New Zealand; ^3^ Gran Sasso Science Institute L'Aquila Italy

**Keywords:** difference‐in‐ difference, disaster risk, managed retreat, mental health, relocation

## Abstract

People are sometimes forced to move, and it is plausible that such relocation involves significant psychological costs. The challenge in identifying the mental health consequences of moving is that most moves are (at least partly) voluntary so that the sample of movers is self‐selected. We focus on a natural experiment, the government‐mandated relocation of some households after all households experienced an exogenous shock. We use this experiment to identify the causal impact of moving on people's mental health, distinguishing between less severe and more severe health conditions, and between individuals with pre‐existing mental health conditions and those without. The event we focus on is the 2011 Christchurch (New Zealand) earthquake, and the consequent decision of the government to relocate about 8000 households from some of the earthquake‐affected areas. We use a comprehensive administrative dataset that includes health records with information on hospital attendance, specialist services, and prescribed medications for (almost) every resident in the city and compare the relocated individuals to those who lived elsewhere in the earthquake‐damaged city. We examine both the likelihood of receiving mental health treatment (the extensive margin), and the intensity of treatment, measured by the number of visits to a clinic or hospital (the intensive margin). We find a statistically significant increase in the likelihood and frequency of receiving treatment for moderate mental health problems among individuals compelled to relocate, when compared to other residents of the earthquake‐affected city who were allowed to remain in situ. This increase persisted to December 2013 for everyone, and remained significant for the elderly to the end of 2018. We found no such increase in health care utilisation for more severe mental health symptoms that required more acute interventions (in clinics or hospitals).

## 1 Introduction

People are sometimes forced to move, and it has often been hypothesized that such relocation involves significant economic, social, and psychological costs. Most obvious is the loss in income potential that arises because of the loss of location‐specific skills that the migrant possessed, and the connections with occupations for which the migrant has trained for. These are much more likely to manifest if the move is long‐distance, and more if it also crosses linguistic, cultural, or administrative borders. Equally, migration also involves significant loss of cultural, linguistic, and social connections (i.e., social capital) that may directly leads to a loss in wellbeing. Again, the longer the distance is, the more likely it is to involve the loss of social capital. Similarly, migration may exact psychological costs that originate from “place attachment” (i.e., the emotional bond to the home), or from other changes that are associated with the process of moving. It is these psychological costs—ones that may manifest in seeking treatment for mental health (the variable we measure)—that we focus on in this paper.

The challenge in identifying the mental health consequences of moving is that most moves are voluntary, that is, based on the individual's (or household's) decision and initiated by them. This self‐selection therefore prevents interpreting differences between observed outcomes in those people who move and those who don't as causally associated with the move itself. Put differently, this selection makes it is impossible to identify any causal impact of such moves. Even after a sudden disaster may destroy a person's house, the house's residents still make a voluntary choice whether to move or stay in situ (even if this decision is constrained differently than in moves that are not triggered by external shocks)—see, for example, Deryugina et al. (2018).

This, however, is not the case if the move is mandated, and is largely random (in the sense that this mandate to move is unlikely to be correlated with any pre‐move characteristics of the movers). We use such a natural experiment, the mandated relocation of some households after an exogenous shock, to identify the causal impact of moving on people's mental health. The event we focus on is the Christchurch (New Zealand) earthquake of February 2011, and the consequent decision of the government to relocate about 8000 households from the affected area, because of a risk of liquefaction. This liquefaction, a geological process that makes the water table rise and soil “liquefy”, made re‐building too costly for the public insurer in the aftermath of the earthquake. Elsewhere in the earthquake‐affected city, even in places that possibly experienced more destruction than in these red‐zoned areas, homeowners were allowed to rebuild in the same place.

The earthquake sequence that peaked with the February 2011 event, and generated this relocation program, started in September 2010, with an earthquake in Darfield, east of the city of Christchurch. This earthquake caused extensive damage to buildings and infrastructure, but few deaths. However, a shallower aftershock that occurred around midday the 22^nd^ of February 2011 was centered much closer to the center of the city and led to much more extensive damage. Most tragically, this earthquake killed 185 people. As some of the deaths occurred in buildings whose structural integrity was damaged in the earlier 2010 earthquake, this event led to a wholesale re‐examination of risk in the city, including a 2‐year cordon of the central business district of the city, widespread demolitions of damaged large office buildings (where the majority of the deaths occurred), and a re‐assessment of the viability of some residential areas.

Most of the city of Christchurch, with around 400,000 people, lies on a very flat area (the Canterbury Plains) close to the east coast of the South Island, with rivers running through it. The center of the city consisted of mostly office and commercial buildings of several stories, and much of the rest of the city was composed of suburban low‐rise homes. To the south‐east of the city center is a peninsula of volcanic origin which contains some of the more expensive suburbs located on steep hillsides. The earthquake mortality was associated almost exclusively with the larger buildings in the central business district (especially with two that collapsed completely), and almost no one died or was injured in the surrounding low‐slung residential neighborhoods which are the focus of our research.

The earthquake caused a rise of the water‐table that led to widespread liquefactions in several areas, especially in residential neighborhoods along the banks of the Avon and Waimakariri Rivers. It also caused some rockfalls, cliff collapses, and slope instability in the volcanic peninsula. Practically, every house in the city incurred at least some damage from the earthquake sequence even though mortality occurred almost only in the center of the city. In June 2011, the government decided to reclassify some of these liquefaction and cliff‐collapse areas as Residential Red Zones (RRZ). In these liquefaction‐ and cliff‐collapse‐prone areas, buildings were found to be uneconomic to repair or too risky to inhabit, respectively. Crucially for our identification, the decision was driven not by the extent of damage, but the viability of future rebuilding there. Homeowners were told that these RRZ areas would no longer be permitted for residential use, and they were expected to move away.

The government offered to compensate the owners by offering to purchase their house and land at their pre‐earthquake values.^1^ This decision ultimately affected 8060 houses and more than 16,000 people across Greater Christchurch (MacDonald and Carlton 2016; C. Nguyen 2020).^2^ As such, this was an exceptionally large relocation program, even when compared to those implemented in much bigger countries (Hino et al. 2017; Mach et al. 2019). A mandated relocation, such as the Christchurch RRZ, may be associated with adverse psychological outcomes for those who are forced to relocate. If these relocations are exogenously determined (i.e., they are unanticipated and uncorrelated with any pre‐event characteristics of the affected individuals) one may be able to determine that these impacts were causal.

The case of the Christchurch RRZ is interesting not only because of its size and “exogenous” origin, but also as relocated residents did not have to move far away and were fully compensated for the value of the property they were surrendering to the government. Because of these aspects, the relocated individuals were not forced to change their employment, nor to sever their links with their community, society, and cultural milieu. Still, relocation may have led to some voluntary changes in employment and some modest reductions in income for some groups (mostly young women—see Hoang and Noy 2023). It is therefore a case where any adverse mental health consequences identified are more likely to be a result of the relocation itself than indirectly due to the impacts it generated (such as by reducing income, wealth, or employment).

We use the Integrated Data Infrastructure (IDI), an administrative database collected and maintained by Statistics NZ (the national statistical agency). The IDI has several advantages. These data are comprehensive, and thus provide large, diverse, and representative samples of the population (very few people are not included), which allows us to reach robust conclusions about sub‐populations we examine. These data also allow for ongoing tracking over time, in a panel set up over an extended period.

The IDI includes administrative health data with information on hospital attendance, community care specialist services, and prescribed medications, and thus holds significant potential for health research. Here, we investigate the impact of the forced relocation on mental health. As we have individual records of practically all the residents of Christchurch, we can further identify these impacts across various sub‐samples including separating samples by gender, age, income, and ethnicity. Finally, we can identify the longer‐run effects of relocation on the RRZ residents, as long as they stayed in New Zealand (and were thus observable in the government's administrative records).

Our investigation is tangentially related to several other literature, though as far as we are aware none has asked the question we ask here, using a causal identification strategy like the one we use here: What are the mental health consequences of being forced to relocate by an exogenous event?^3^ The epidemiological literature has examined the mental health consequences of disasters before, mostly asking whether Post Traumatic Stress Disorder (PTSD) and Major Depressive Disorder (MDD) are generated by these disasters. It generally finds that PTSD and MDD often occur jointly in those affected by catastrophic events—see the reviews by Emily and Galea (2014), Rataj et al. (2016) and Beaglehole et al. (2018).^4^ Most of this research relies on before‐after comparisons of prevalence rates in affected communities, without typically an ability to control for other time variant effects and without access to individual records. Even when individual records are available, they are usually aggregated spatially and prevalence rates are then compared across affected and un‐affected areas.^5^

Another strand focused specifically on forced relocations due to disasters, but these were not because of an exogenous government mandate. These relocations happened because of the destruction experienced or the loss of income sources caused people to move (e.g., Fussell and Lowe 2014). As such, it was impossible to separate the impact of relocation from the destruction and loss wrought by the disaster itself, and not surprisingly, this literature largely finds adverse mental health impacts.

A different literature looks at relocations in other contexts, though most of these relocations were voluntary. In voluntary cases, selection issues prevent any clear causal assignment of the relocation to measurable mental health outcomes. One exception is a randomised control trial, the Moving to Opportunity (MTO) Study, conducted during the 1990s in five U.S. cities. Kling et al. (2007) and Osypuk et al. (2012) examined the psychological impacts of this trial, which targeted specifically vulnerable populations living in poor neighborhoods and offered them the opportunity to move, after a lottery‐based assignment, to wealthier neighborhoods.^6^ The MTO's findings may not be, therefore, generalizable beyond this specific context of a lottery win to a better neighborhood—though it clearly confirms some of the benefits of “moving to opportunity.” Ludwig et al. (2013), another paper focusing on this experiment, concludes that adults mental health improved when they moved to a higher SES neighborhood. Harding et al. (2021), more recently, summarizes much of the finding from the MTO trial and attempts to reconcile it with observational data that finds significant additional benefits to moving (beyond what is identified in the trial). Much of this literature is described in Chyn and Katz (2021).

Another random assignment of relocation from acute poverty, in this case, in India, has reportedly generated a significant severing of social and community ties and may have inadvertently led to a significant deterioration of people's wellbeing (Barnhardt et al. 2017). This example resembles some of our findings with respect to mental health, in spite of the very different context.

Another group of papers analyzed the post‐earthquake prevalence of prescribing of psychological medications in Christchurch. These papers largely looked at the difference in prevalence rate of various treatments in the earthquake‐affected areas and compared these to unaffected areas elsewhere in New Zealand. These include: Beaglehole et al. (2020) which used psychiatric prescription medication data for the elderly and youth, respectively; Beaglehole et al. (2019) which used a longitudinal survey; and Greaves et al. (2015), Hogg et al. 2014 which used Ministry of Health data. The closest investigation to ours is Hogg, Kingham, Wilson, and Ardagh (2016a), which examined the impact of relocation on individuals affected by the Christchurch earthquake, when compared to individuals who did not relocate. As noted earlier, this type of investigation suffers from significant selection bias, since the relocation was partly voluntary, even if it followed from the earthquake‐wrought destruction. The direction of bias in this case is unclear, as it could be that mental health challenges were related with the decision to move, or alternatively were associated with a reluctance to move.

To summarize, our goal is to identify the mental health impacts of the forced relocation of individuals in the aftermath of the Christchurch earthquakes, noting that this relocation was mostly within the city to other neighborhoods with little significant socio‐economic differences from the neighborhoods the relocated left behind. The next section provides a brief overview of the Residential Redzone Program (RRZ) while Section 3 details the data we use, Section 4 describes their statistical characteristics and Section 5 outlines our econometric approach. Section 6 discusses our results, and the last section concludes with some caveats and discusses the relevance of our findings to the climate adaptation discussions of managed‐retreat programmes.

## 2 Residential Red Zone (RRZ) Program

The relocation program implemented after the 2011 Christchurch earthquake was designed to manage the movement of residents from areas that were judged to be either unsafe or uneconomic to maintain or to reconstruct. The Canterbury Earthquake Recovery Authority (CERA), a government body, was tasked with overseeing the program. It offered financial compensation to owners of insured RRZ properties; renters did not get any support. Property owners were given the option to accept either the full 2007 rateable value for land, buildings, and fixtures, with any residual insurance claims assigned to the government, or the full 2007 rateable land value only, allowing them to retain insurance claims on buildings and fixtures (if these were damaged in the earthquakes).^7^ The government made this initial offer to households in the Ōtākaro‐Avon River corridor in June 2011, followed by the same offer to the Red Zoned households in the Waimakariri District and the Port Hills. This offer was accepted by the majority of affected residents. By mid‐2015, approximately 7000 houses had been removed from the river corridors and around 1000 from the eastern parts of Kaiapoi and its small coastal settlements (Hogg, Kingham, Wilson, and Ardagh 2016b). Most households moved fairly quickly after they have made their choice and signed the offer documents. CERA, the government body in charge, was eager to resolve this as quickly as possible, demolish and clear and remaining houses, and start the process of consultation about the long‐term use of these areas.

The RRZ program was designed to provide financial relief and facilitate relocation from areas deemed unsustainable for continuing habitation. While the offer was widely accepted, it was not mandatory, and property owners had the option to refuse it. The government, however, stated that utility services to those choosing to remain (electricity, water, and sewage) will be disconnected; in effect making this offer compulsory (or at least perceived as such). The terms of the offer were standardized, and there was no scope for individual negotiation. The government offered homeowners the two options: (1) The Crown Option: full buyout of building and land. (2) The Insurance Option: buyout of the land, with the homeowner retains any residual claim against their insurer for any damage. The final date for accepting these offers was December 10, 2015; but the vast majority accepted these offers much quicker than that. By the deadline, 7724 of 8060 property owners in the residential red zone had accepted one of the governments offers; 1695 homeowners had accepted the Crown Option, and 6029 properties had chosen the Insurance Option.

Very few of the remaining property owners were still negotiating with the government after the deadline, but most were for properties that had no insurance at the time of the earthquake (many were vacant lots that could not be insured). Owners of uninsured properties were initially told by the government that they will get no compensation at all. But after a bruising legal fight, the government relented and offered the same Crown Option offer (described above).

**FIGURE 1:**
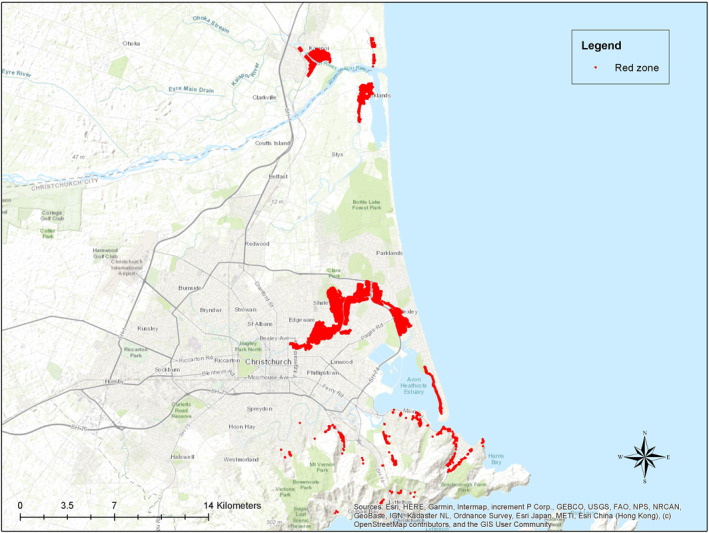
Residents of the red zone areas in Canterbury. The Waimakariri corridor is the area at the top of the map. The Avon river corridor is the large area in the middle of the map, while the Port Hills houses are scattered around the peninsula to the south‐east of the city. See also Appendix 1. *Source:* The authors and Land Information New Zealand.

Most RRZ residents chose to relocate within Christchurch, many electing to purchase homes in new subdivisions that were established during the city's reconstruction. As a result, the population of the Ōtākaro‐Avon River Corridor statistical area, which encompassed the majority of the red zone, saw a significant decline. In 2006, the population was 10,386, dropping to 1263 in 2013, and decreasing to 99 by 2018 (Cloke et al. 2023). Figure 1 maps the RRZ areas while Appendix 3 describes the timeline in detail.

Given our focus on mental health, we note that the RRZ program focused exclusively on the relocation and compensation of affected homeowners, and did not include any dedicated health care services (including no additional mental health services). The RRZ program also did not include any specific support for renters who resided in the RRZ, as it was the owners of their homes that received the government compensation.^8^ The broader recovery efforts in Christchurch did involve significant health care support, which included emergency medical care and ongoing health services to maintain community health (Adams‐Hutcheson 2015). It is plausible that the increased focus on general health and well‐being during the recovery period could have influenced the frequency of GP visits observed in our study, but it would have been equally available to both the “treated” and the “control” individuals in our dataset as long as they stayed in the Canterbury Region.

We note that some people from the “control” group (earthquake‐affected individuals not residing in the RRZ) also chose to move in the aftermath of the earthquake. Removing these “movers” from our sample would have inserted a selection bias into the control group, as they are likely different from those controls who chose to stay. Fortunately for our estimation, not that many people chose to move (about 18% within the 4‐year time period ‐ well in line with standard rates of relocation in New Zealand—see Table A4). This number is non‐negligible, but including this “mover” group in our control group biases our estimates in an unknown direction. If the people who chose to move in the control group have better mental health than those who chose to stay from that control group, then our results would be biased downward. If, on the other hand, it is those who were more likely to experience mental health challenges who chose to move, it would bias our results upward. Put differently, if we assume that the voluntary relocations (in the control group) are not too dissimilar to involuntary ones (in the treatment group), in terms of their consequences for mental health, then our results would be biased upward if we were to exclude the voluntary movers from our control sample. This may well be the case. In any case, we interpret our results narrowly, and argue that they pertain to mandatory relocations, as we do not know if there are fundamental differences in voluntary versus mandatory moves, in terms of their impact on mental health.

## 3 Data

### 3.1 Data Sources

#### 3.1.1 Mental Health Data

The healthcare system in New Zealand operates across three main levels: community, primary, and secondary services. Community services provide accessible support, often informal and without the need for a doctor's appointment, these are less well recorded, and typically deal with more routine services. The primary services are the typical entry point into the public healthcare system. Although patients can directly access secondary services, it is more common for referrals to come from primary services. In this study, mental health data are extracted from primary and secondary service records. We use mental health data records between 2009 and 2018 as this time period is the common overlap between the different administrative sources we use. We use these data to test our hypothesis: That being a resident in the RRZ at the time of the Christchurch earthquakes may be associated with a later change in mental health, expressed as anxiety, mood, depression, or eating disorders.

The data come from 3 sources: (1) The pharmaceutical collection contains claim and payment information from pharmacies for subsidized dispensing of prescriptions. It is not possible to identify if people actually take the medicines, but our aim is to identify the onset of symptoms that generated the prescription, not the efficacy of treatment. Unsubsidized dispensing is not included in this data and is unknowable. This pharmaceutical data has been linked to the IDI administrative records by the Ministry of Health and PHARMAC since 2005. (2) The National Minimum DataSet (NMDS) includes reported discharges from hospitals for inpatients and day patients from all of the publicly funded hospitals (by far most people are treated in the public hospital system). Privately funded hospital events have been excluded from this dataset in the IDI because not all privately funded hospitals submit their data to the Ministry of Health continuously. For our study, NMDS data collected from July 2008 onwards were used. (3) Program for the Integration of Mental Health Data (PRIMHD) includes specialist services from all publicly funded service providers (thus excluding privately funded services). PRIMHD is a set of national mental health data collected by a Ministry of Health on service use and diagnoses. Data is collected from District Health Boards (BHDs) and non‐governmental organizations (NGOs) that provide government‐funded mental health and addiction treatments. PRIMHD data are also available in the IDI from July 2008.

In this study, mental health indicators used to identify mental health care utilisation (doctor/clinic appointments and prescriptions) include anxiety, mood, depression, eating disorder, and substance abuse. We do not examine mental health indicators that may not be caused by relocation but are more likely to be genetically determined, such as dementia and attention‐deficit hyperactivity disorder. Mental health markers were categorized into two groups: less severe cases comprised individuals who visited a general practice doctor (GP) and received a prescription, as indicated by pharmaceutical data, while severe cases included individuals who were hospitalized or utilized publicly‐funded specialized services, as indicated by a combination of the NMDS and PRIMHD data. For individuals with less severe mental health issues, 98% were associated with mood disorders, while the remaining 2% were linked to substance abuse. In the severe level group, eating disorders represented less than 3%, whereas both mood disorders and substance abuse accounted for almost a half of cases each.

#### 3.1.2 Demographic and Socioeconomic Data

We obtain demographic characteristics, including date of birth, gender, and ethnicity, from census data. Ethnicity is classified into six groups: European, Māori, Pacific, Asian, Middle Eastern/Latin American/African (MELAA), and Other, following the Statistics New Zealand standard for ethnicity. In this study, we examine two ethnic groups: Māori, the indigenous Polynesian people of New Zealand, and non‐Māori. Age is calculated by subtracting the year of birth from year of study, which ranges from 2009 to 2018. Education records are sourced from the Ministry of Education data. New Zealand educational qualifications are categorized into 10 levels, ranging from 1 to 10. The lowest levels (1–3) correspond to certificates, while level 10 corresponds to a doctoral degree. Detailed information on qualification levels and types can be found in Appendix 5.

Data on deprivation are obtained using the New Zealand Deprivation Index (NZDep) which is based on the deprivation characteristics of “meshblocks”. For instance, the NZDep13 integrates 2013 census data on various factors, including income, employment, qualifications, communication, support, living space, transport, and home ownership, into a single measure of relative socioeconomic deprivation. The NZDep is presented in deciles, with each decile representing approximately 10% of the meshblocks in New Zealand. Specifically, Decile 1 represents areas with the least deprived scores, while Decile 10 represents areas with the most deprived scores. In this paper, we use NZDep2006 from the 2006 census data, which is the most recent census from before the earthquakes.^9^

### 3.2 Integrating Multiple Data Sources to Construct the Estimation Dataset

The IDI locates each observed individual to a meshblock.^10^ Those meshblocks that were classified as part of the RRZ were used to identify the list of individuals who resided in these areas between March 2010 and June 2011 and were therefore subject to the mandatory relocation requirement. Of the 348 meshblocks that were part of the RRZ program, we also distinguished between meshblocks that were wholly within the RRZ (261 meshblocks) and those where the boundary of the RRZ passed through the meshblock thus making identifying whether an individual was subject to a relocation mandate less accurate (87 meshblocks). We only classified these individuals residing in the 261 meshblocks wholly within the RRZ as our “treatment” group. The controls were identified from all the individuals residing elsewhere in the Territorial Authorities of Christchurch and Waimakariri (but also excluding the 87 RRZ boundary meshblocks). Children aged 0–15 years were excluded due to their low incidence of mental health care utilization and their reliance on parental decisions regarding access to medical services (see Ardagh et al. 2012).

Next, we linked all the region's meshblocks to earthquake housing damage maps (based on residential public insurance claims—for a description of this data, see Beaglehole et al. 2020). This allows us to identify the degree of damage experienced in each individual's meshblock, and thus restrict our control group only to those individuals whose meshblocks experienced similar earthquake intensities and housing damage levels to the RRZ residents. We defined housing damage as the ratio of the total value of building damage claims assessed by the Earthquake Commission to the property's value, based on local council valuations. If no claims were recorded, the ratio was zero. This ratio was chosen because it reflects damage relative to property value, minimizing socioeconomic bias. The average damage level per dwelling in each meshblock was assigned to individuals in both the RRZ and the control group based on their meshblock residence.

We categorized the damage experienced in each meshblock throughout Christchurch into three damage groups. In the Red Zone, these were: for group A—light damage (< 6%; 12 meshblocks); group B—moderate damage: (6%–14%; 93 meshblocks), group C—severe damage (≥ 15%; 159 meshblocks). As there were few people who resided in group A meshblocks in the RRZ (around 4%), we excluded them from both the treatment and control group. These thresholds, while arbitrary, are based on C. N. Nguyen and Noy (2020). In Appendix 4, we show there are only minimal differences, in terms of mental healthcare utilisation, between the moderate and severe damage groups.^11^

Among the RRZ movers, 82% relocated within the Canterbury region and most of the others relocated to other regions in New Zealand. Moreover, 66% relocated within the same territorial authority ‐ Christchurch City or the adjacent Waimakariri District, depending on the location of their residence (see Appendix 1, Table A1, A2 to A3). For those RRZ people who moved further away, the top three destinations were, Otago, Auckland, and Wellington—these are the neighboring region to the south of Canterbury, and the two largest cities in New Zealand, respectively (see Teng et al. 2017). We note that people in the control group moved as well; but we choose to keep them in the control group as removing these movers‐by‐choice will insert a selection bias into the control group sample. But keeping them, the control group includes everyone in the dataset who resided in similarly damaged meshblocks during the immediate time before the earthquake.

We examine whether the socioeconomic conditions, as measured by the NZ Deprivation Index 2006, differ between RRZ areas and areas where residents have relocated to. We compared two groups: RRZ meshblocks and relocated meshblocks—that is, those meshblocks into which RRZ individuals chose to relocate to within Christchurch (Test 1); and RRZ meshblocks and all relocated meshblocks within New Zealand (Test 2). Given that the NZDep ranks range from 1 to 10, we applied the Wilcoxon Rank‐Sum Test. The null hypothesis posits that there is no difference in the NZDep between Redzone areas and relocated areas, while the alternative hypothesis suggests a difference between these groups. Both Test 1 and Test 2 revealed test values of 0.3 and 0.47, respectively, with *p*‐values greater than 0.1 (0.58, 0.49, respectively). These results indicate that there is no statistically observable difference in the NZDep or social economic condition between the RRZ areas and relocated areas. These comparisons suggest that the relocation impacts we observe do not arise because the new peers (in the new relocated neighborhoods) were fundamentally different from the peers in the RRZ neighborhoods—unlike the case in the MTO experiments described above. In Appendix 2, we also show that the share of ownership of houses was similar between the RRZ and control groups (66% and 58%, respectively). We note that only owners received compensation as part of the relocation process, but renters also did not experience any financial loss associated with the loss of the property title.^12^

Using the lists of individuals from the treatment and control groups, we aggregated data for 6‐month intervals starting from January 2009 to December 2018. Pharmaceutical data, NMDS, and PRIMHD data are collected daily, but for consistency, we grouped these data into 6‐month intervals. We then combined the lists of individuals from the treatment and control groups with the pharmaceutical data, NMDS, and PRIMHD data to compile all mental health treatment events every six months from January 2009 to December 2018. The 2009 start date was based on data availability.

Subsequently, we segmented the dataset into two distinct groups: (1) Individuals without any mental health records from January 2009 to June 30, 2011 (prior to the introduction of the RRZ program). (2) Individuals with a pre‐RRZ mental health diagnosis made between January 2009 and June 30, 2011. We have a balanced panel dataset of the RRZ and control groups from 2009 to 2018, with 6‐month intervals for the recording of mental health care utilisation events. This dataset allows us to observe whether new instances of mental health treatments emerged following the implementation of the RRZ program among those previously unaffected and whether existing mental health conditions recurred among those with prior mental care records. The specific questions we aim to investigate are these.

1. Did involuntary relocation, because a person resides in the RRZ, increased the likelihood of a new mental health diagnosis for them, when compared to individuals who similarly experienced the damaging earthquake but were not mandated to move?
2. Did involuntary relocation increase the frequency of mental health treatments for RRZ individuals who already had observable mental health problems, when compared to similar individuals who also had pre‐RRZ mental health problems, and had similarly experienced the damaging earthquake, but were not mandated to move?

## 4 Descriptive Statistics of the Data

Table 1 describes the statistics for the data we use. The RRZ residents and the control group share a similar average age. The age distribution between the two groups is also very similar, with 19% aged 16–24, 37% aged 25%–44%, and 43% aged over 44 years old. Within the RRZ group, 52% are women, 11% are Māori, and 64% were enrolled in tertiary education at some point. Similarly, the control group comprises 51% women, 9% Māori, and 63% with tertiary education qualifications. We conducted a balance test to assess the variation in characteristics between the RRZ and control groups (column 5). There are no systematic differences in age, gender, the final year they attended school, frequency of visiting a GP for mental health concerns, or total income in the first half of 2011. While ethnicity, tertiary education enrollment, pre‐existing mental health conditions (from January 1, 2009 to June 30, 2011), and qualification level exhibit statistically significant differences between the groups, these variations are very small.

**TABLE 1:** Descriptive statistics.

Variables	Control		Redzone		Balance tests
	*N*	Mean or % (SD)	*N*	Mean or % (SD)	
	(1)	(2)	(3)	(4)	(5)
*Age in 2011*	108,132	44 (19)	7821	43 (18)	*F* = 1.06
*Age group in 2011*					*χ*2 = 14.34^***^
16–24	20,727	19%	1464	19%	
25–44	40,191	37%	2907	37%	
45–64	30,468	28%	2334	29%	
65+	16,743	15%	1119	14%	
*Gender*					*χ*2 = 2.85
Men	52,659	49%	3729	48%	
Women	55,473	51%	4092	52%	
*Tertiary education enrollment*					*χ*2 = 7.05^***^
No	39,918	37%	2769	36%	
Yes	68,214	63%	5052	64%	
*Last school year*	62,052	1992 (15)	4662	1992 (15)	*F* = 1.92
*Ethnicity*					*χ*2 = 29.31^***^
Non‐ Māori	97,956	91%	6939	89%	
Māori	10,176	9%	882	11%	
*Pre‐existing less severe mental disorder*					*χ*2 = 23.43^***^
No	91,932	85%	6489	83%	
Yes	16,203	15%	1332	17%	
*Average number of times visiting a GP*	108,132	0.52 (3.8)	7821	0.57 (3.7)	*F* = 1.43
*Pre‐existing severe mental disorder*					*χ*2 = 17.90^***^
No	97,605	90.3%	6942	88.8%	
Yes	10,524	9.7%	879	11.2%	
*Total income ($NZD)*	93,525	14,127 (17,505)	7068	13,970 (12,111)	*F* = 0.67

*Note:* SD refers to the standard deviation. Age is in 2011. Total income is stated in 2018 NZD, the number of times visiting a GP was determined during the first 6 months of 2011; Pre‐existing mental disorders were identified from January 1st, 2009, to June 30th, 2011. Age in 2011 is calculated by subtracting the year of birth from 2011. Less severe cases were identified as those who visited a GP, while severe cases were identified as those who were admitted to the hospital or used specialist services.

*p < 0.1.

**p < 0.05.

*** *p* < 0.01.

During Jan 2009 to June 2011‐ the two and a half years before the RRZ program was announced, we identified the individuals who were treated for mental health. This group includes about 10% of the entire cohort in both the control and red zone groups that had pre‐existing but moderate mental health issues, while only about 2% had been facing pre‐existing severe mental health challenges.

**FIGURE 2:**
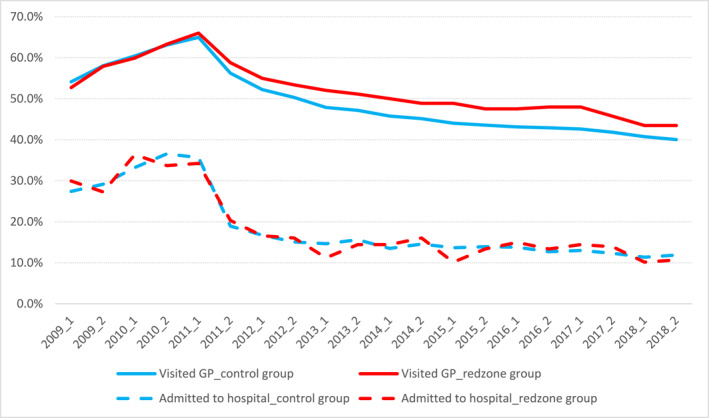
Rates of mental health treatments for individuals with pre‐existing conditions. Individuals with pre‐existing mental health conditions are defined as those who had any mental health issues, including visits to the GP, hospital admissions, or use of specialized mental health services, from January 1, 2009, to June 30, 2011.

**FIGURE 3:**
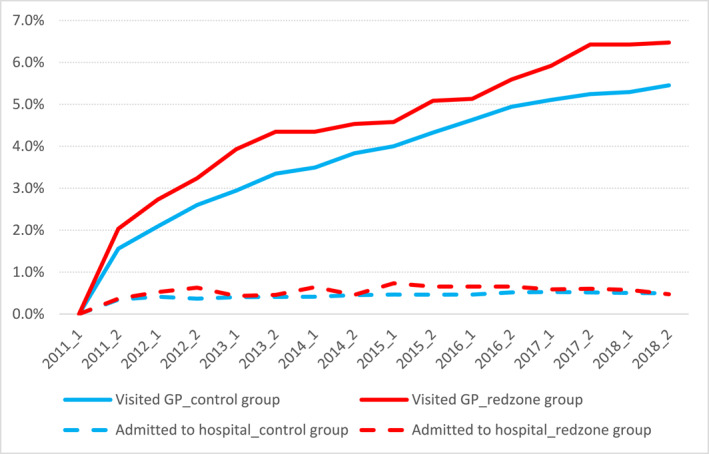
Rates of mental health disorders among individuals without pre‐existing conditions. Individuals without pre‐existing mental health conditions are defined as those who did not have any mental health issues, including visits to the GP, hospital admissions, or use of specialized mental health services, from January 1, 2009, to June 30, 2011.

Figures 2, 3 show the percentages of individuals with and without pre‐existing mental health treatment. The top lines illustrate GP visits, while the bottom lines show hospital admissions or special services for both the red zone and control groups. In Figure 2, rates increased from January 2009 to mid‐2011, then declined, with RRZ residents experiencing a slower decrease. Figure 3 indicates higher rates of mental health issues in the RRZ group, rising rapidly every 6 months post‐relocation until 2013, then stabilizing in 2014–2015, suggesting more severe short‐term effects of relocation. The similar figures for the separate damage‐level groups (moderate and severe damage) are in Appendix 4. For both those with and without pre‐existing treatment, severe mental health issues were infrequent, with no observable significant difference between treatment and control groups. Overall, the numbers of individuals admitted to hospitals or those utilizing specialist services are small. We note that the number of individuals with pre‐existing severe mental health issues accounted for approximately 2% of the entire cohort in both the control and red zone groups. There are consequently instances where the rates in the RRZ group fell below those of the control group, but these data should be interpreted with caution due to the very small sample size.

## 5 Econometric Methodology

Our approach to assessing the impact of involuntary relocation on mental health, including both the occurrence and severity of issues, at the individual level, focuses on comparing RRZ residents to their neighbors (identified as the control group in Section 3) after the introduction of the RRZ program at the end of June 2011 (a difference‐in‐difference strategy).

Given that the presence of mental health issues during a given period is a binary outcome at the individual level, we employ a logistic regression model. Since the severity of issues—defined by the frequency of visits to a GP for less severe conditions, or admissions to the hospital, or utilization of special services for severe conditions—is represented by counts, we utilize a Poisson regression model for the intensive margin. We use generalized linear model (GLM):

$$g\left(µ\right)=\beta_{0}+\beta_{1}\;{Treat}_{i}+\beta_{2}\;{Post}_{t}+\beta_{3}\;{Treat}_{i}*{Post}_{t}{\;+\;\beta }_{4}X_{it}+e_{i}+u_{it}$$

To estimate the average impact from 2011 to 2018, we use the following equation:

(1) $$g\left(µ\right)=\beta_{0}+\beta_{1}\;{Treat}_{i}+\beta_{2}\;{Post}_{t}+\beta_{3}\;{Treat}_{i}*{Post}_{t}+\beta_{4}X_{it}+e_{i}+u_{it}$$

To examine the dynamic impact over time in detail, we estimate equation:

(2) $$g\left(µ\right)=\beta_{0}+\beta_{1}{\text{Treat}}_{i}+\sum_{t}\beta_{t}{\text{Post}}_{t}+\beta_{3}\left({\text{Treat}}_{i}\times {\text{Post}}_{t}\right)+\beta_{4}X_{it}+e_{i}+u_{it}$$

For.

- Logistic regression: ($g\left(µ\right)=\log(\frac{P\left(Y_{it}=1\right)}{1-P\left(Y_{it}=1\right)})$
- Poisson regression: ($g\left(µ\right)=\log(Y_{it}$)

Where: $Y_{it}$ denotes whether person *i* had received a medical intervention for a mental health issue (prescription drugs or specialist services) in every half‐year period *t*, or it denotes the number of medical interventions for mental health events for person *i* in period *t.* ${Treat}_{i}$ is an indicator variable equal to 1 if person *i* lived in the RRZ areas before June 2011. ${Post}_{t}$ is a dummy variable equal to 1 if periods from July 2011 to Dec 2018. $X_{it}$ includes age, gender, highest education level, ethnicity, and earthquake damage level. As the RRZ program was announced at the end of June 2011, we combine data for each 6 months period (January‐June and July‐December); we include individual fixed effect ($e_{i}$) to account for time‐invariant unobservable factors. Since in this type of longitudinal data, the observations of the same person may be correlated over time, so are the error terms for each person ($u_{it}$). Our preferred specification, therefore, clusters standard errors at the individual level.^13^

The odds and incident rate ratios are defined as: $Odds=\frac{p}{1-p}$, where *p* is the probability of experiencing mental health issues; and one‐*p* is the probability of not experiencing mental health issues. The Incident Rate Ratio $\left(IRR\right)=\frac{events}{person\;time};$ where *events* refer to the number of visits to a GP for moderate mental health conditions. For severe mental health conditions, the number of admissions to the hospital or usage of specialist services are counted. *person time* refers to the number of 6 months periods. Each $\beta_{t}$ in (2) captures the effects of the treatment at different time periods, allowing us to see how the impact changes over time.

We are interested in estimating the treatment effect of relocation, which we refer to as TE:

(3) $$\left(TE\right)=\frac{\frac{Odds/IRR\;\left[mental\;health|redzone,\;postRRZ\;program\right]}{Odds/IRR\;\left[mental\;health|redzone,\;preRRZ\;program\right]}}{\frac{Odds/IRR\;\left[mental\;health|control,\;postRRZ\;program\right]}{Odds/IRR\;\left[mental\;health|redzone,\;preRRZ\;program\right]}}$$

We can calculate the mental health odds of people in the RRZ and the control group, before and after the implementation of the RRZ program, while adjusting our estimates for differences in various factors across people and periods. The difference‐in‐difference in Equation (1) estimation give us the $\beta_{3}$ = ln(TE). We thus report the exp ($\beta_{3}$) as the odds or IRRs of mental health for individuals living in RRZ areas relative to the odds or IRRs for individuals living in non RRZ areas before the earthquakes. Additionally, we calculate the marginal effect to provide the absolute change in probability or count number. We initially estimate effects across all cohorts, then stratify into subgroups based on whether these individuals were with or without pre‐existing recent mental health treatment. Subsequently, within each of these groups, we estimate effects for various demographic strata, including gender (men and women), age categories (16–24, 25–44, 45–64, and 65+), ethnicity (Māori and non‐Māori), tertiary education (individual with and without tertiary education experience), and housing damage levels (moderate and severe).

## 6 Estimation Results

### 6.1 Treatments for Moderate Mental Health Problems

We report the relocation effects separately for moderate mental health conditions and more severe ones. For each, we present the relocation effects on the likelihood of experiencing mental health problems estimated with a logit model (i.e., the extensive margin) and the intensity of mental health problems as measured by the number of treatments (i.e., the intensive margin). For OR, a value greater than (less than) one indicates that the odds of experiencing a mental health issue for the RRZ group exceed (are below) those of the control group. An OR of exactly one indicates no difference in the likelihood of having a mental health issue between the RRZ and control groups. For the IRR, compared to the control group, the incidence rate (or the expected count) of visits to the GP or admission to the hospital for the RRZ group increases by the factor expressed by the IRR (if IRR > 1), while values less than one indicate a decrease in the incidence rate.

All regression table results show the estimated treatment effect obtained from a separate regression for the relevant sample or sub‐sample. Thus, the first row and first column in Table 2 shows the impact of being an RRZ resident on the likelihood of obtaining prescription medication (for mental health issues) for the full sample. In this table, the odds ratio provides insight into the relative change in odds, while the marginal effect offers an absolute change in probability. Thus, RRZ residents had 22% higher odds of receiving such prescription, and the probability of experiencing moderate mental health problems requiring pharmaceutical interventions increases by 0.8% points than similarly earthquake‐affected residents of Christchurch.

**TABLE 2:** The likelihood of receiving moderate (pharmaceutical) mental health treatments.

	OR‐ full sample		OR‐ without previous mental health treatment		OR‐ with previous mental health treatment	
	Logit odds ratio (1)	Marginal effects (2)	Logit odds ratio (3)	Marginal effects (4)	Logit odds ratio (5)	Marginal effects (6)
All observations	1.222**** (0.071)	0.0189 (0.006)	1.153** (0.073)	0.0027 (0.001)	1.349**** (0.098)	0.0026 (0.001)
Women	1.178** (0.086)	0.0169 (0.008)	1.077 (0.090)	0.001 (0.001)	1.354**** (0.124)	0.0037 (0.001)
Men	1.297*** (0.123)	0.0214 (0.008)	1.285** (0.132)	0.0046 (0.002)	1.336** (0.161)	0.0014 (0.001)
16–24	1.098 (0.138)	0.0033 (0.004)	1.036 (0.132)	0.00093 (0.003)	1.240 (0.183)	0.0429 (0.29)
25–44	1.023 (0.093)	0.0007 (0.003)	0.964 (0.09)	−0.00033 (0.0009)	1.109 (0.12)	0.0088 (0.009)
45–64	1.039 (0.117)	0.0039 (0.012)	1.055 (0.133)	0.0002 (0.0005)	1.022 (0.149)	0.00002 (0.0002)
65+	2.478**** (0.189)	0.033 (0.003)	1.8**** (0.280)	0.012 (0.003)	3.597**** (0.385)	0.113 (0.010)
Māori	0.890 (−0.143)	−0.0074 (0.01)	0.898 (0.152)	−0.003 (0.005)	0.829 (0.154)	−0.0123 (0.013)
Non‐Māori	1.270**** (0.079)	0.0239 (0.006)	1.192*** (0.080)	0.0032 (0.001)	1.436**** (0.113)	0.0025 (0.001)
Tertiary education	1.055 (0.073)	0.0025 (0.003)	1.016 (0.076)	0.0003 (0.001)	1.162**** (0.09)	0.007 (0.004)
No tertiary education	1.608**** (0.169)	0.0765 (0.021)	1.513**** (0.177)	0.0098 (0.003)	1.785**** (0.126)	0.069 (0.009)
Moderate housing damage (group B)	1.052 (0.117)	0.0047 (0.010)	0.993 (0.119)	−0.00012 (0.002)	1.232 (0.171)	0.0017 (0.001)
Severe housing damage (group C)	1.303**** (0.096)	0.025 (0.008)	1.285*** (0.103)	0.0055 (0.002)	1.367**** (0.127)	0.0036 (0.001)

*Note:* Each observed coefficient is obtained from a separate regression. Standard errors are in parentheses.

Abbreviation: OR: odds ratio.

*p < 0.1.

** *p* < 0.05.

*** *p* < 0.01.

**** *p* < 0.001.

Table 2 (column 1) shows the results of the logistic OR regressions. Compared to their neighbors, RRZ residents were more likely to receive prescriptions for mental health problems. This was true especially for the elderly, non‐Māori, individuals who had no tertiary education, and those who experienced severe housing damage. While both men and women, when estimated separately, had an increase likelihood of being prescribed, the effect of being in the RRZ seems to be larger for men (this difference is statistically significant). Conversely, no effects were observed for individuals younger than 65, for Māori, and for those with tertiary education, or for those whose houses sustained less damage (group B).

Overall, RRZ residents had 22% higher odds of experiencing mental health issues after relocation compared to those who didn't relocate. This adverse effect was observed for both women (18%) and men (30%). Most acutely, elderly individuals were 2.5 times more likely to experience mental health issues after relocation when compared to elderly individuals in the other earthquake‐affected areas.

Table 2 (columns 3–6) reports the separate estimation of the effects of relocation for individuals with and without previous mental health treatment records. Specifically, columns 3–4 present the findings for individuals who did not have a recent history of mental health treatments. Generally, among individuals without a pre‐earthquake recent history of mental health treatments, those residing in the RRZ areas during the time of the earthquake were associated with a 1.15 times higher likelihood of receiving mental health medications compared to the control group (statistically significant). Among this group, the elderly (80%) and those without tertiary education (51%) experienced the most significant adverse observable mental health problems from their forced relocation. Results for individuals with a recent history of mental health treatments are presented in columns 5–6. These effects are similar to those observed for the entire cohort in columns 1–2, with larger coefficients, as many of the treated individuals in the post‐earthquake period had also received treatments before (see Figures 2, 3).

As shown in Table 3, the Poisson intensity‐margin regressions indicate that only the elderly group was impacted by relocation in terms of the frequency of mental health treatments. Specifically, on average from 2011 to 2018, RRZ's elderly individuals had 29% higher rate of mental‐health‐related visits to a GP or an average increase of 0.25 GP visits per 6‐months period compared to the control group.

**TABLE 3:** The frequency of receiving moderate (GP visits) mental health treatments.

Variables	IRR‐ full sample		IRR‐ without previous mental health treatment		IRR‐ with previous mental health treatment	
	Poisson rate ratio (1)	Marginal effects (2)	Poisson rate ratio (3)	Marginal effects (4)	Poisson rate ratio (5)	Marginal effects (6)
All observations	1.037 (0.087)	0.036 (0.084)	1.071 (0.082)	0.068 (0.076)	1.045 (0.097)	0.044 (0.093)
Women	1.079 (0.111)	0.076 (0.103)	1.042 (0.101)	0.041 (0.097)	1.109 (0.128)	0.103 (0.116)
Men	0.962 (0.141)	−0.039 (0.147)	1.181 (0.161)	0.166 (0.136)	0.919 (0.132)	−0.084 (0.143)
16–24	1.150 (0.143)	0.140 (0.110)	1.077 (0.151)	0.074 (0.140)	1.105 (0.157)	0.100 (0.142)
25–44	0.932 (0.086)	−0.070 (0.093)	0.876 (0.123)	−0.131 (0.140)	0.966 (0.088)	−0.035 (0.092)
45–64	0.966 (0.187)	−0.035 (0.194)	0.984 (0.153)	−0.015 (0.155)	0.949 (0.201)	−0.052 (0.212)
65+	1.289*** (0.114)	0.254 (0.088)	2.013**** (0.339)	0.699 (0.168)	1.287*** (0.112)	0.252 (0.087)
Māori	0.712 (0.259)	−0.340 (0.364)	1.129 (0.273)	0.121 (0.241)	0.738 (0.259)	−0.304 (0.352)
Non‐Māori	1.079 (0.088)	0.076 (0.082)	1.114 (0.091)	0.108 (0.082)	1.085 (0.101)	0.082 (0.093)
Tertiary education	1.046 (0.125)	0.045 (0.120)	0.935 (0.092)	−0.066 (0.098)	1.100 (0.146)	0.095 (0.133)
No tertiary education	0.988 (0.104)	−0.012 (0.106)	1.443** (0.180)	0.366 (0.125)	0.94 (0.100)	−0.062 (0.107)
Moderate housing damage	1.124 (0.278)	0.117 (0.248)	0.928 (0.154)	−0.074 (0.166)	1.231 (0.339)	0.208 (0.275)
Severe housing damage	0.966 (0.076)	−0.035 (0.078)	1.217** (0.114)	0.196 (0.093)	0.932 (0.074)	−0.070 (0.080)

*Note:* Each observed coefficient is obtained from a separate regression. Standard errors are in parentheses.

Abbreviation: IRR: Incidence rate ratio.

*p < 0.1.

** *p* < 0.05.

*** *p* < 0.01.

**** *p* < 0.001.

Additionally, we performed regression analyses using a linear probability model (Appendix 6), and for different mental health prescription medications (Appendix 7). Results from the linear probability model are very similar, and compared with those in the control groups, people in RRZ had a 23% higher likelihood to be treated for mood and anxiety and the difference was statistically significant (*p* < 0.001). No statistically significant findings in substance use treatment were identified.

Results from Tables 2 and 3 indicate that the elderly are the most affected age group. Subsequently, we conducted regression analysis exclusively for the elderly to investigate the factors influencing their mental health (Table 4). For the elderly (age > 65), being a woman, non‐Māori, lacking tertiary education, and having severe damage to the house (group C) were the sub‐groups most affected by relocation with a likelihood 2.5–2.7 times higher of experiencing mental health issues compared to the control group (Table 4, column 1). It is only for the elderly Māori that we did not observe a significant difference in the likelihood of experiencing mental health issues between the RRZ and the control groups.

**TABLE 4:** The likelihood and frequency of receiving treatment for the elderly.

Variables	OR‐ likelihood		IRR‐ frequency	
	Logit odds ratio (1)	Marginal effects (2)	Poisson odds ratio (3)	Marginal effects (4)
Women	2.730**** (0.586)	0.0369 (0.004)	1.265** (0.129)	0.235 (0.102)
Men	2.194*** (0.586)	0.0297 (0.005)	1.347* (0.235)	0.298 (0.175)
Māori	1.098 (0.792)	0.0035 (0.003)	1.583** (0.358)	0.459 (0.226)
Non‐Māori	2.526**** (0.428)	0.0350 (0.003)	1.284*** (0.115)	0.250 (0.089)
Tertiary education	1.780* (0.624)	0.0205 (0.06)	1.327* (0.205)	0.283 (0.1549)
No tertiary education	2.750**** (0.527)	0.0391 (0.003)	1.278** (0.130)	0.245 (0.1019)
Moderate housing damage	2.171** (0.689)	0.0294 (0.006)	1.388* (0.256)	0.328 (0.184)
Severe housing damage	2.597**** (0.551)	0.0353 (0.004)	1.288** (0.144)	0.253 (0.111)

*Note:* Each observed coefficient is obtained from a separate regression. Standard errors are in parentheses.

Abbreviations: IRR: Incidence rate ratio, OR: odds ratio.

* *p* < 0.1.

** *p* < 0.05.

*** *p* < 0.01.

**** *p* < 0.001.

**FIGURE 4:**
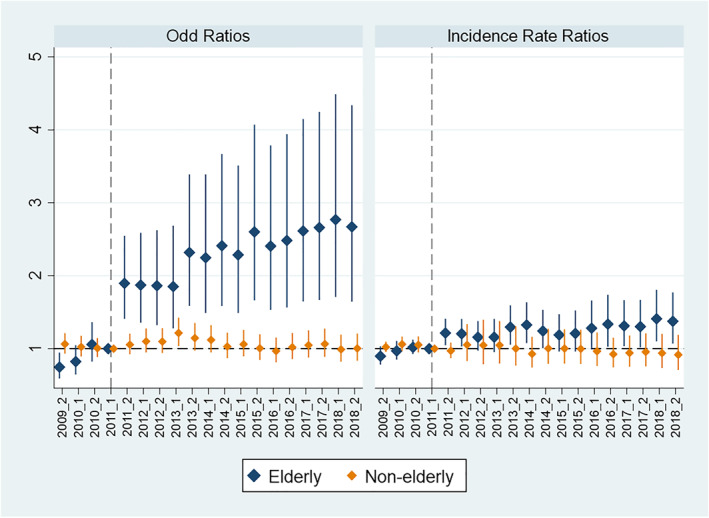
The dynamic effects odd ratios (left panel) and incidence rate ratio (right panel).

In addition to the estimation of the average relocation effect over the post‐relocated periods, we also are interested in the dynamic effects over time, and therefore estimate our specifications for different points in time since July 2011. For this, we estimated an “event study” specification, incorporating the interaction of treatment and time for each period. Figure 4 presents the estimated dynamic impacts. It is obvious from this analysis that while the extensive‐margin impact on the non‐elderly is statistically significant, this is identified only through 2013. Much larger impacts are observed for the elderly, in both the extensive and intensive margins, and these persist until the end of our dataset at the end of 2018, more than 7 years after the earthquake, and at least 5 years after the forced relocation away from the RRZ areas.

### 6.2 Treatments for Severe Mental Health Problems

We estimate the likelihood of being treated for more severe mental health problems and the incidence rate ratios number of time which people visited the hospital or used specialist psychiatric services in Table 5. Overall, relocation did not significantly affect severe mental health treatments in terms of both likelihood and frequency. We do observe a weaker statistically significant effect for the elderly and individuals whose houses were in the less damaged group (group B), while no statistically significant effect was found for other groups. Overall, we note that many fewer people were treated in the hospitals or in specialist services for mental health problems, so the statistical power of our analysis is significantly lower.

**TABLE 5:** The likelihood and frequency of receiving treatment for severe mental health problems.

Variable	OR‐ full sample		IRR‐ full sample	
	Logit odds ratio (1)	Marginal effects (2)	Poisson rate ratio (3)	Marginal effects (4)
All observations	1.108 (0.124)	0.0255 (0.0280)	1.180 (0.043)	0.165 (0.129)
Women	1.038 (0.167)	0.008 (0.0355)	1.205 (0.061)	0.186 (0.050)
Men	1.179 (0.185)	0.038 (0.037)	1.143 (0.226)	0.134 (0.198)
16–24	1.113 (0.284)	0.025 (0.059)	1.378 (0.445)	0.321 (0.323)
25–44	0.926 (0.183)	−0.011 (0.029)	1.037 (0.198)	0.036 (0.191)
45–64	1.083 (0.218)	0.016 (0.041)	1.193 (0.236)	0.176 (0.198)
65+	1.554** (0.375)	0.003 (0.001)	1.662** (0.464)	0.508 (0.279)
Māori	1.137 (0.302)	0.024 (0.050)	0.920 (0.272)	−0.083 (0.296)
Non‐Māori	1.098 (0.136)	0.023 (0.030)	1.254 (0.179)	0.226 (0.143)
Tertiary education	0.964 (0.137)	−0.008 (0.031)	1.206 (0.175)	0.187 (0.145)
No tertiary education	1.386** (0.254)	0.049 (0.033)	1.129 (0.258)	0.121 (0.229)
Moderate housing damage	1.507** (0.394)	0.101 (0.065)	1.774** (0.402)	0.573 (0.226)
Severe housing damage	0.981 (0.129)	−0.0039 (0.026)	0.924 (0.156)	−0.079 (0.169)

*Note:* Each observed coefficient is obtained from a separate regression. Standard errors are in parentheses.

Abbrevations: IRR: Incidence rate ratio, OR: odds ratio.

*p < 0.1.

** *p* < 0.05.

***p < 0.01.

****p < 0.001.

## 7 Discussion

Several studies have documented elevated levels of mental health challenges among populations that relocate. In these cases, however, the decision to relocate is often a combination of external pressures combined with household choice. It is thus difficult to causally ascribe the mental health conditions to the move. This study contributes a causal identification, as it uses a “natural experiment”—a relocation that was mandated and was, to a large extent, randomly assigned (in terms of households' characteristics) to different neighborhoods in the city of Christchurch, as it was predicated on risk that was not recognised before the earthquake. As we use individual‐administrative data for the entirety of the population residing in the city, we are thus able to investigate the impact of a mandated relocation on different sub‐populations and the dynamic impacts on these sub‐populations over time.

Specifically, we look at the aftermath of the Christchurch 2011 earthquake and differentiate between those individuals who were mandated to relocate under the RRZ “managed retreat” program, and other people in Christchurch who were similarly affected by the earthquake but were not forced to relocate in the earthquake's aftermath (though their houses may have suffered similar amounts of damage). By linking mental health treatment services to individual demographic characteristics, we were able to isolate the impact of these mandated relocations from other confounding factors.

We found a statistically significant increase in the likelihood and frequency of receiving treatment for moderate mental health problems among individuals compelled to relocate, when compared to other residents of the city whose houses were not situated in the RRZ areas and were thus not compelled to relocate. This effect persisted from July 2011 to December 2013 and remained significant across all the examined period to 2018 for the elderly. Thus, we identified both short‐term and long‐term adverse mental health impacts. While a prior study—Hoang and Noy (2023), using different methodology and different data—primarily examined short‐term effects, our study provides more causal evidence confirming the enduring adverse impact of the relocation, and identifies this enduring impact specifically for the elderly.

We note that the RRZ program did not have any health support services component, as this would have been a possible explanation for our finding of increase in the utilisation of mental health services (and prescriptions). It is, however, possible that those who were forced to relocate were thus also disconnected from their primary health care providers or may have found it more difficult to make appointments with them because of the relocation, for other reasons (longer travel distance, time constraints associated with the relocation, etc.). If this is the case, then our estimates could be viewed as a minimum, as it is likely that with easier access there would also have been more utilisation of services.

Even though the identified relocation effects were statistically significant, the numbers of new mental health patients remained small. This is possibly due to the strong community cohesion observed in many post‐disaster situations, as 82% of relocations occurred within the Canterbury region.^14^ Additionally, generous government compensation was provided to RRZ residents, so relocation was unlikely to involve very significant material hardship; even though it did lead to some small declines in earned income for young women, but only for them (Hogg, Kingham, Wilson, and Ardagh 2016b).

It is noteworthy that the indigenous people of Aotearoa New Zealand, the Māori, were not observed to experience a statistically significant impact with respect to both the frequency and likelihood of their interactions with the mental health treatment system post‐relocation. One possible explanation is the enhanced resilience of this population, but this could also be because of the lower likelihood of Māori to seek or to receive treatment for mental health problems compared to non‐Māori more generally. Less access for Māori for many types of health services has been documented in many different contexts and involve several possible reasons. The most common explanation focuses on the documented distrust in the health system among Māori arising out of the historically different (and often inferior) treatment provided to Māori by the public health system. This points, again, to a weakness in our empirical approach. What we observe is not the mental health conditions themselves, but the utilisation of treatment for them. In as much as many mental health challenges go undiagnosed and/or untreated, we are unable to capture and investigate the dynamic incidence of untreated mental health challenges, and their connections to mandated relocation for the various affected population groups in the city.

### 7.1 The RRZ as a Managed Retreat Program

From an adaptation policy perspective, the RRZ program was a standard “managed retreat” program, not dissimilar to such programs that are motivated by the changing frequency and intensity of extreme weather events due to climate change. Hoang and Noy (2023) argue that “if future policies and plans for managed retreat are to be implemented successfully, a great deal of further work is required since they have neglected important psychological, symbolic, and particularly emotional aspects of healthy human habitats and that a failure to address this crucial qualitative aspect of relocation may fundamentally undermine wider policy and planning initiatives on adaptation to climate change.” (p. 509).

The importance of mental health as a component of the process of managed retreat has been recognised already at least 15 years ago, but remarkably we have not found any attempt to quantify whether Agyeman et al. (2009)'s concern about the mental health consequences of managed retreat motivated by climate change is indeed quantitatively relevant—see for example the review in Agyeman et al. (2009). In short, “how big is this psychological aspect?” is an important question, as without it, it is difficult to see how a full consideration of the costs and benefits of a decision to relocate in a program of managed retreat can be appropriately weighed.

In the context of climate change, two types of managed retreat programmes have generally been implemented: Slow and deliberate pre‐disaster anticipatory retreat, and post‐disaster retreat that is initiated after a catastrophic event led to physical destruction and increased saliency of the risk. The first is considered the gold standard in the managed retreat literature, but one that is much more difficult and costly to implement, In contrast, post‐disaster managed retreat is less expensive as the destroyed homes need to be reconstructed somewhere, anyway. Post disaster programmes are also easier to implement as the increased salience of the risk makes them politically less fraught (Solecki and Friedman 2021). The RRZ is essentially an example of a post‐disaster managed retreat program, and that is reflected in the (relatively) easy acceptance it received from the affected communities. The policy literature has suggested that more deliberate and carefully planned retreats are easier psychologically (e.g., Arnold et al. 2023). However, this hypothesis has never been tested in practice, and has just been assumed to be true (Dundon and Abkowitz 2021). We are unsure if it is true, but our investigation may be seen as a first step in constructing an informed answer to this question. The next step in obtaining a fuller answer is the answer to the second question: Would the same kind of stress caused by the RRZ managed‐retreat program we examined manifest in an anticipatory relocation program?

### 7.2 Limitations and Caveats

Some limitations of our study are worth noting. First, we had no access to the relocation contracts that people signed and the amount of compensation they received—this amount was mostly determined by the value of their house, if they owned their residence (Dannenberg et al. 2019). Renters, in contrast, did not receive any assistance—though we are unable to separately identify renters and owners. Our focus is on the mental health impact of people when they are forced to relocate, but it is conceivable that the impact may be very different for renters and homeowners, and for owners according to the amount of compensation received and the terms in which this compensation is provided.^15^ Unfortunately, with our data, we cannot identify these differences and their relevance to mental health.

Second, we identify the location of individuals at every period at the meshblock level based on the reported meshblock in the administrative data. This information is sourced from various datasets, including health data, census data, and education data. Stats NZ consolidates these sources, along with the location and time they report. We prioritize the location with the most recent reported date within each period. We acknowledge certain limitations, including the infrequent updating of addresses in the administrative data. Additionally, we lack individual data on housing situations, such as whether individuals were living in temporary accommodations or remained in their homes while awaiting to find a new home to relocate to.

Furthermore, there is a large literature that ties social capital (bonding, bridging, and linking social connections—see C. Nguyen 2020) to post‐disaster economic outcomes, including related to mental health. Presumably, the loss of bonding social capital may be very important for mental health, since in this case neighborhoods were scattered because of the RRZ policy and thus at least some social ties were severed. It is likely that the breakdown of social ties, especially of significance to the elderly, is a mechanism that explains our results with the much stronger effects for the elderly. However, our data does not allow us to measure social capital directly, so we cannot verify this conjecture. An alternative explanation, of course, is that it is the emotional ties to one's home and immediate physical environment (e.g., a local park or the river) that has led to the impact we identified. We cannot differentiate between these two possibilities—between the severing of social ties to people, and emotional ties to place.

## 8 Conclusion

This study contributes to the understanding of the mental health impacts of forced relocations, using a natural experiment from the Christchurch 2011 earthquake. By analyzing individual‐level administrative data, we isolated the effects of the mandated relocation program, providing evidence of increased mental health care utilisation among relocated individuals. Our findings indicate a statistically significant increase in moderate mental health symptoms among those forced to relocate, persisting from mid‐2011 to 2018 among the elderly. These results highlight both short‐term and long‐term adverse effects of these relocations.

Notably, our study reveals that individuals without prior mental health conditions are more vulnerable to developing issues post‐relocation, with the elderly showing particularly higher susceptibility. This underscores the need for targeted mental health support for relocated populations, support that was not specifically available at the time for the RRZ population. From a policy perspective, our findings stress the importance of considering mental health impacts in the design of managed retreat programs, which are in turn a crucial component of climate change adaptation strategies more broadly.

## Conflicts of Interest

The authors declare no conflicts of interest.

## IDI Disclaimer

The results in this paper are not official statistics. They have been created for research purposes from the Integrated Data Infrastructure (IDI) managed by Statistics New Zealand. Access to the anonymized data used in this study was provided by Statistics New Zealand in accordance with security and confidentiality provisions of the Statistics Act 1975. The findings are not Official Statistics. The results are based on tax data supplied by Inland Revenue to Statistics NZ under the Tax Administration Act 1994. This tax data must be used only for statistical purposes, and no individual information may be published or disclosed in any other form or provided to Inland Revenue for administrative or regulatory purposes. Any person who has had access to the unit record data has certified that they have been shown, have read, and have understood Section 81 of the Tax Administration Act 1994, which relates to secrecy. Any discussion of data limitations or weaknesses is in the context of using the IDI for statistical purposes and is not related to the data's ability to support Inland Revenue's core operational requirements. The opinions, findings, recommendations, and conclusions expressed in this paper are those of the author(s), and not Statistics NZ.

## Figures and Tables

**FIGURE A1:**
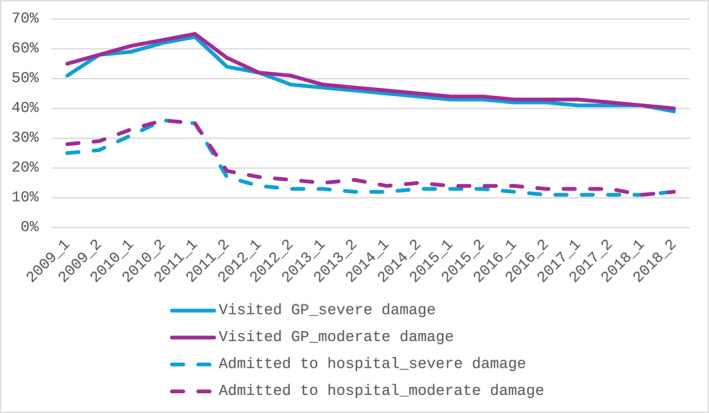
Rates of mental health disorders among individuals with pre‐existing conditions.

**FIGURE A2:**
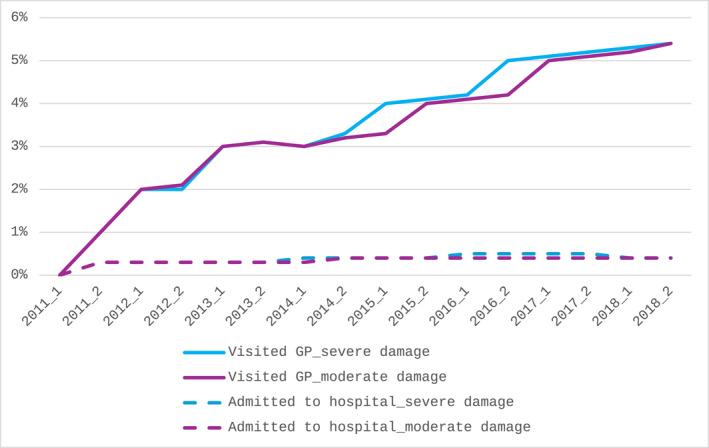
Rates of mental health disorders among individuals without pre‐existing conditions.

## Data Availability

The data that support the findings of this study are available from Statistics New Zealand. Restrictions apply to the availability of these data, which were used under license for this study. Data are available from https://www.stats.govt.nz/ with the permission of Statistics New Zealand.

## Endnotes

^1^
Much more detail about the exact government offer is available in Aldrich (2012).

^2^
By April 2015, approximately 7800 property owners (over 96 percent of the eligible group) had accepted the government's offer to sell their properties to the Crown, most had already settled their claimed compensation, and nearly all had already relocated.

^3^
Hoang and Noy (2020) also looked at a sudden exogenously driven relocation associated with a volcanic event in Iceland, but their focus was on income and educational attainment.

^4^
For a pertinent example on the post‐disaster mental health prevalence among Japanese survivors of the 2011 Great East Japan Earthquake see Nakamura et al. (2022) and (2021).

^5^
Several studies have found an inverse relationship between age and adverse mental health outcomes in adults following natural disasters. Hikichi et al. (2017), for example, reported that middle‐aged disaster victims exhibit the highest risk for distress and subsequent mental health issues.

^6^
A similar exogenous relocation from poorer to higher SES neighborhoods is investigated in Norris et al. (2002).

^7^
The rateable values are used for property tax assessments. They are updated every 3 years.

^8^
In the data we have access to, we cannot distinguish between homeowners and renters.

^9^
The NZDep estimates relative socioeconomic deprivation for areas, not individuals. It cannot be used to assess changes in absolute deprivation over time, as the classification is relative.

^10^
Meshblocks are the smallest geographically unit identified by Statistics NZ. They are like Census tracts in the US context, but significanly smaller (about 100 people on average, per meshblock).

^11^
Alternatively, we could have assigned a level of damage for each individual, based on the meshblock average damage, as we do not know the actual damage experienced by each individual in the IDI. This would have inserted a lot of mis‐classification into our data, we therefore chose to divide the sample into three levels of damage.

^12^
Content insurance was available to everyone through the public insurer (the Earthquake Commission) irrespective of ownership status.

^13^
In STATA, we use the clogit command to run the fixed effects logistic regression model for panel data when the outcome variable is binary, and xtpoison command to run the fixed effects regression model when the outcome variable is a count variable.

^14^
For details about the destinations of those relocated, see Haltiwanger et al. (2024). For examples for this general observation about the strength of social capital after disasters, see Hoang and Noy (2023).

^15^
For details about these contracts, see Solnit (2010). For details about the importance of the contractual details about people's wellbeing in the RRZ case, see C. Nguyen (2020).

## Appendix 1 Moving Behaviors after the RRZ Program

**TABLE A1:** Proportions of people categorized in RRZ over time.

Year in RRZ areas	Percentages (%)
2011	77.3
2012	22.4
2013	0.3
Total	100

**TABLE A2:** Destinations of RRZ Residents by territorial authority (TA).

Destinations	Percentages (%)
Same territorial authority	66.2
Different TA, still in the Canterbury region	16.0
Different TA, outside the Canterbury region	17.8

**TABLE A3:** Destination by move date of RRZ Residents.

Moved year	Destination (region)	Percentages (%)
	(1)	(2)
2011	Stayed in Canterbury	82.2
	Auckland	4.4
	Otago	2.7
	Wellington	1.9
	Marlborough	1.5
	Bay of plenty	1.3
	Tasman	1.1
	Westcoast	1
	Others	4
2012	Stayed in Canterbury	89.6
	Auckland	2.4
	Otago	1.7
	Wellington	1
	Others	5.3
After 2013	Stayed in Canterbury	89.4
	Auckland	2
	Otago	3
	Wellington	1
	Others	4.6

**TABLE A4:** Percentages of stayers/movers in the control group by housing damage level until the end of 2015.

	Stayers	Movers
Moderate housing damage (group B)	66%	15%
Severe housing damage (group C)	16%	4%

By 2015, the majority of people in the control group remained at the same location as before the red zone program (around 82%). Among those who moved, interestingly, the percentage of people who experienced moderate housing damage was higher than that of people who experienced severe housing damage. It should be noted that 95% of housing in New Zealand is covered by insurance. This suggests that those in the control group might have relocated by their own choice, although the exact reasons behind their decisions are unknown.

## Appendix 2 The Socioeconomic Situation of the Control and Redzone Groups Prior to Treatment

We examine the ownership of dwellings using the 2006 Census data, which is the closest available Census pre‐treatment. We identified households in the redzone and control meshblocks and calculated the percentage of households in these areas that owned a house. However, it should be noted that the Census data are not linked in the IDI, so there is no way to incorporate this variable into the main dataset. We do not have more recent data than the 2006 Census for the period up to 2011.

Interestingly, the percentage of redzone residents who owned a house was higher than that of the control group (66% vs. Fifty‐eight percent). This suggests that redzone residents may not be relatively low‐SES and more vulnerable to mental health issues. Therefore, their mental health issues could be attributed to the relocation program rather than their socioeconomic status Table A5.

**TABLE A5:** The percentages of house ownership among control and redzone groups in 2006.

	Own a dwelling	Not own	Other
Control group	58%	31%	11%
Redzone group	66%	24%	10%

## Appendix 3 Residential Redzone Program Timeline

March 29, 2011, Establishment of the Canterbury Earthquake Recovery Authority (CERA).

April 18, 2011, Passing of the Canterbury Earthquake Recovery Act 2011.

June 23, 2011, Announcement of the Government purchase offer to owners of insured red zoned residential properties in the Christchurch flat areas. Property owners could accept.

- Hundred percent of the 2007 rateable value for land, buildings, and fixtures on the property (any residual insurance claim was then assigned to the Government), or
- Hundred percent of the 2007 rateable land value only (and all insurance claims on buildings and fixtures on the property were retained by the owners).

August 18, 2011, Announcement that this same offer would be extended to owners of the 940 insured red zoned residential properties in Waimakariri District and also in the Port Hills south‐east of Christchurch.

August 19, 2011, Mailout of purchase offers to the first 3000 owners of eligible red zoned properties.

September 13, 2012, Announcement of the Government purchase offers for owners of vacant land, and commercial and uninsured properties, in the flat‐land residential red zone.

August 26, 2013, Decision of the High Court that the government had no prerogative power to create the RRZ, and that the Canterbury Earthquake Recovery Minister had not followed correct statutory procedure.

December 3, 2013, The Court of Appeal decided that the decision to red zone parts of Greater Christchurch was lawful, and that there was a rational basis for distinguishing between insured and uninsured properties, but that the 50% offer was not in line with the Recovery Act.

March 13, 2015, Decision of the Supreme Court that the Government's September 2012 decisions relating to uninsured RRZ property owners and to vacant residential landowners were not lawfully made.

April 21, 2015, Announcement of the decision to develop a Recovery Plan, allowing public commentary on the Government offers to owners of red zoned commercial, vacant and uninsured properties. Eventually, uninsured properties were offered the same as insured ones.

## Appendix 4 Mental Health Rates by Housing Damage Level and Pre‐existing Mental Health Conditions for the Control Group.

The rate lines for moderate and severe damage are close for both GP visits and hospital admissions, indicating no significant differences in mental health consequences among damage levels. This can be explained by the compensation from insurance, which covers 95% of houses in New Zealand (Figure A1 and A2).

## Appendix 5 New Zealand Educational Qualification Levels

The New Zealand Qualifications Framework (NZQF) is a framework based on outcomes, described in terms of knowledge, skills and attributes, and their application. New Zealand qualifications are based on need, outcomes, flexibility and collaboration. This approach is intended to provide a simple structure for qualifications and programmes. Qualifications are designed to identify the underpinning skills, knowledge and attributes graduates need to perform a range of roles across a broad context. The table below describes different levels and corresponding qualification types.

**Table:**

Level	Qualification types
10	Doctoral degree
9	Master's degree
8	Postgraduate Diplomas and certificates
	Bachelor Honors degree
7	Bachelor's degree
	Graduate Diplomas and certificates
5–6	Diplomas
1 to 4	Certificates

*Source:* The New Zealand qualification framework (https://www.nzqa.govt.nz/assets/Studying‐in‐NZ/New‐Zealand‐Qualification‐Framework/requirements‐nzqf.pdf).

## Appendix 6 Robustness Checks.

(see, Table A6, Table A7, Table A8)

**TABLE A6:** Linear probability model (LPM) regression results.

	The likelihood of receiving mental health treatments	The frequency of receiving mental health treatments
All observations	0.0097**** (0.0023)	0.0275 (0.0482)
Women	0.0095*** (0.0035)	0.0636 (0.0765)
Men	0.00980*** (0.0030)	−0.0120 (0.0564)
16–24	0.0086* (0.0045)	0.0274 (0.0256)
25–44	0.0034 ((0.0037)	−0.0239 (0.0458)
45–64	0.0031 (0.0042)	−0.0132 (0.1450)
65+	0.0396**** (0.0075)	0.2270*** (0.0825)
Māori	−0.0042 (0.0064)	−0.1600 (0.200)
Non‐Māori	0.0114**** (0.0025)	0.0494 (0.0480)
Tertiary education	0.0042 (0.0028)	0.0445 (0.0635)
No tertiary education	0.0191**** (0.0041)	−0.0860 (0.0711)
Moderate housing damage (group B)	0.0838 (0.0044)	0.0838 (0.157)
Severe housing damage (group C)	0.0120**** (0.0029)	−0.0121 (0.0426)
With pre‐mental health conditions	0.0408*** (0.0097)	0.1380 (0.274)
Without pre‐mental health conditions	0.0079**** (0.0018)	0.0210 (0.0160)

*Note:* Each observed coefficient is obtained from a separate regression. Standard errors are in parentheses.

* *p* < 0.1.

***p* < 0.05.

*** *p* < 0.01.

**** *p* < 0.001.

**TABLE A7:** OLS regression results.

	(1)	(2)
Post*treatment	0.0041**** (0.0017)	0.0038** (0.0018)
Age	0.0005**** (0.00001)	0.0005**** (0.00001)
Gender (ref: Males)		
Females	0.023**** (0.0004)	0.023**** (0.0004)
Ethnicity (ref: Non‐Māori)		
Māori	−0.1164**** (0.00065)	−0.124**** (0.0007)
Pre‐existing mental health conditions (ref: Without conditions)		
With conditions	0.4482**** (0.0010)	0.4482**** (0.0011)
Housing damage group (ref: Moderate housing damage)		
Severe housing damage	0.0017*** (0.0005)	0.0023**** (0.0005)
NZDep2006		0.0006**** (0.00008)
*R*‐square	0.29	0.29

*Note:* Standard errors are in parentheses.

* *p* < 0.1.

** *p* < 0.05.

*** *p* < 0.01.

**** *p* < 0.001.

**TABLE A8:** OLS regression results with interactions.

	(1)	(2)
Post treatment	0.0135**** (0.002)	0.0119** (0.0018)
Post*treatment Gender (ref: males)	−0.0023 (0.002)	−0.0023 (0.002)
Post treatment ethnicity (ref: non‐Māori)	−0.0199**** (0.0029)	−0.0139**** (0.0032)
Post treatment pre‐existing mental health conditions (ref: without pre‐existing mental health conditions)	−0.1899**** (0.0045)	−0.147**** (0.0050)
Post treatment housing damage levels (ref: severe housing damage)	−0.0087**** (0.0024)	−0.011**** (0.0026)
Control for age, gender, education, ethnicity, housing damage levels	Yes	Yes
Control for NZDep2006	No	Yes

*Note:* Standard errors are in parentheses.

* *p* < 0.1.

** *p* < 0.05.

*** *p* < 0.01.

**** *p* < 0.001.

## Appendix 7 Mental Health Status of the RRZ Residents and Control Group by Indicators.

Table A9

**TABLE A9:** Percentages of people with less severe mental health symptoms.

Period	Control		Red zone	
	Mood and anxiety	Substance use	Mood and anxiety	Substance use
	(1)	(2)	(3)	(4)
2009_1	8.1%	0.1%	8.9%	0.2%
2009_2	8.6%	0.1%	9.8%	0.1%
2010_1	9.0%	0.1%	10.1%	0.3%
2010_2	9.4%	0.1%	10.7%	0.2%
2011_1	9.7%	0.2%	11.2%	0.2%
2011_2	9.7%	0.2%	11.6%	0.1%
2012_1	9.5%	0.2%	11.6%	0.2%
2012_2	9.7%	0.2%	11.8%	0.2%
2013_1	9.6%	0.2%	12.0%	0.1%
2013_2	9.9%	0.2%	12.2%	0.3%
2014_1	9.8%	0.2%	12.0%	0.2%
2014_2	10.0%	0.2%	12.0%	0.2%
2015_1	9.9%	0.2%	12.1%	0.2%
2015_2	10.2%	0.2%	12.2%	0.2%
2016_1	10.3%	0.2%	12.3%	0.2%
2016_2	10.6%	0.2%	12.7%	0.2%
2017_1	10.7%	0.2%	13.0%	0.2%
2017_2	10.7%	0.2%	13.1%	0.2%
2018_1	10.5%	0.2%	12.7%	0.2%
2018_2	10.6%	0.2%	12.7%	0.2%

Table A10

**TABLE A10:** Rates of people with severe mental health by indicators.

Period	Control			Red zone		
	Mood and anxiety	Substance use	Eating disorder	Mood and anxiety	Substance use	Eating disorder
	(1)	(2)	(3)	(4)	(5)	(6)
2009_1	48.9%	49.4%	1.7%	56.8%	37.8%	5.4%
2009_2	49.0%	49.0%	2.0%	59.0%	38.5%	2.6%
2010_1	49.1%	48.6%	2.4%	52.9%	45.1%	2.0%
2010_2	56.1%	41.8%	2.1%	47.9%	52.1%	0.0%
2011_1	50.1%	47.2%	2.7%	66.0%	29.8%	4.3%
2011_2	50.6%	46.0%	3.4%	60.0%	40.0%	0.0%
2012_1	50.4%	46.7%	2.9%	51.9%	46.2%	1.9%
2012_2	53.1%	45.0%	1.8%	52.9%	43.1%	3.9%
2013_1	49.4%	49.0%	1.6%	51.6%	45.2%	3.2%
2013_2	46.7%	52.4%	0.9%	47.6%	47.6%	4.8%
2014_1	47.3%	50.8%	1.8%	42.2%	53.3%	4.4%
2014_2	48.3%	49.4%	2.3%	45.9%	54.1%	0.0%
2015_1	51.6%	46.4%	2.0%	48.2%	51.8%	0.0%
2015_2	51.4%	46.0%	2.6%	54.3%	45.7%	0.0%
2016_1	47.9%	50.0%	2.1%	40.0%	56.0%	4.0%
2016_2	49.6%	48.9%	1.4%	48.9%	51.1%	0.0%
2017_1	48.3%	50.3%	1.4%	53.3%	44.4%	2.2%
2017_2	51.2%	47.2%	1.5%	45.7%	54.3%	0.0%
2018_1	46.8%	52.2%	1.0%	50.0%	50.0%	0.0%
2018_2	47.9%	50.3%	1.8%	34.4%	65.6%	0.0%

Table A11

**TABLE A11:** The likelihood of experiencing less severe mental health disorders by indicators.

Variables	OR mood and anxiety		OR substance use	
	Logit odds ratio (1)	Marginal effects (2)	Logit odds ratio (3)	Marginal effects (4)
All cohort	1.231**** (0.066)	0.0084 (0.0023)	0.633* (0.129)	−0.0002 (0.0001)
Women	1.176** (0.079)	0.0066 (0.0030)	0.843 (0.236)	−0.0001 (0.0002)
Men	1.326*** (0.115)	0.0092 (0.0033)	0.514* (0.134)	−0.0006 (0.0003)
16–24	1.133 (0.129)	0.0045 (0.0047)	0.112*** (0.041)	−0.0006 (0.0001)
25–44	1.018 (0.085)	0.0006 (0.0036)	0.825 (0.206)	−0.0002 (0.0004)
45–64	1.053 (0.108)	0.0017 (0.0039)	0.706 (0.212)	−0.0003 (0.0003)
65+	2.503**** (0.363)	0.0346 (0.0062)	0.966 (0.472)	−0.0001 (0.0002)

*Note:* Standard errors are in parentheses.

Abbreviation: OR: odds ratio.

* *p* < 0.1.

** *p* < 0.05.

*** *p* < 0.01.

**** *p* < 0.001.
